# High Dose Ilaprazole/Amoxicillin as First-Line Regimen for* Helicobacter pylori* Infection in Korea

**DOI:** 10.1155/2016/1648047

**Published:** 2016-06-19

**Authors:** WonGun Kwack, YunJeong Lim, ChiYeon Lim, David Y. Graham

**Affiliations:** ^1^Department of Internal Medicine, Dongguk University Ilsan Hospital, Dongguk University College of Medicine, Goyang 10326, Republic of Korea; ^2^Department of Biostatistics, Dongguk University Ilsan Hospital, Dongguk University College of Medicine, Goyang 10326, Republic of Korea; ^3^Department of Medicine, Michael E. DeBakey Veterans Affairs Medical Center and Baylor College of Medicine, Houston, TX, USA

## Abstract

*Objective*. The eradication rate of* Helicobacter pylori* (*H. pylori*) following standard triple therapy has declined over the past few decades. This study has determined whether high dose dual therapy (PPI and amoxicillin) is adequate for eradicating* H. pylori* in Korea.* Methods*. This was an open-labeled study of* H. pylori* infected treatment-naive patients. Subjects received dual therapy for 14 days: ilaprazole 40 mg tablets given twice a day and amoxicillin 750 mg tablets given 4 times a day. At the end of the therapy, the subjects visited the clinic to confirm compliance and monitor for any side effects. Subjects visited again after 4–6 weeks to confirm* H. pylori* status through a urea breath test.* Results.* The cure rate of* H. pylori* was 79.3% (23 of 29) (95% confidence interval: 61.6–90.2) in the intention-to-treat analysis and 82.1% (23 of 28) in the per-protocol analysis. Compliance rates were high (96.6%) and side effects were minimal and tolerable.* Conclusion.* A high dose of ilaprazole + amoxicillin was ineffective as the first-line therapy for eradicating* H. pylori* in Korea. Future studies should focus on intragastric pH measurements and assess amoxicillin resistance.

## 1. Introduction

*Helicobacter pylori (H. pylori)* has been related to a range of gastrointestinal (GI) diseases, such as peptic ulcers, gastric adenocarcinoma, and chronic gastritis [1]. Peptic ulcers are considered to be an infectious disease, and eradicating the causative microbe is a cure. Many studies have reported that* H. pylori* is etiologically related to mucosa-associated lymphoid tissue (MALT) lymphoma and gastric cancer [2–4]. Elimination of* H. pylori* induces the regression of MALT lymphomas and decreases the occurrence of metachronous relapse, following the removal of early gastric cancer by endoscopic resection [5, 6].

Until recently, a proton pump inhibitor (PPI) and the antibiotics clarithromycin and amoxicillin/metronidazole, the so-called standard triple therapy, was used as the first-line eradication therapy for* H. pylori* infection, worldwide [7–10]. Unfortunately, the efficacy of this standard triple regimen has decreased over the past decades, which is thought to be due in large part to increasing antimicrobial resistance. The declined effectiveness of standard triple therapy was also confirmed in a recent Korean study, using meta-analysis. The overall success rate was 74.6% in an intention-to-treat (ITT) analysis and 82% in a per-protocol (PP) analysis [11]. Indiscriminate antibiotics use for the treatments of upper respiratory infections, urinary tract infections, and nonbacterial diseases, such as the common cold, may cause resistance-associated* H. pylori* eradication failure.

The high dose dual regimen, as an alternative first-line medication, is comprised of a high dose PPI and amoxicillin to which* H. pylori* has shown relatively low resistance [12]. Some studies that revealed an increasing effectiveness of a high dose and/or more frequent PPI and amoxicillin administration also reported eradication rates greater than 90% [13, 14]. Despite the advantage of high doses, however, eradication rates vary among studies, and several studies have reported that the CYP2C19 genotype may affect the eradication rate [15, 16]. It is generally thought that high dose PPI therapy is required to reliably increase gastric pH to 6 or above [17, 18]. In addition, data regarding the efficacy, side effects, and patient adherence to the new therapy is scarce in Korea. Therefore, this study was designed to demonstrate whether or not high dose PPI, plus amoxicillin, is efficient as the first-line treatment for* H. pylori* and elucidate compliance and side effects of this therapy.

## 2. Subjects and Methods

### 2.1. Subjects

Eligible subjects were 20–80 years of age. Candidates were consecutive, treatment-naive subjects who underwent upper GI endoscopy with proven* H. pylori* based on Giemsa stained histological findings,* H. pylori* IgG serology, or the rapid urease test (CLO test; Kimberly-Clark Co., Draper, UT, USA) performed at Dongguk University Ilsan Hospital, Korea, between January 8, 2015 and May 8, 2015. Subjects who consented were recruited into the study and their demographic information, including age, sex, underlying gastric disease, smoking, and alcohol consumption, was also collected.

Subjects who fulfilled 1 or more of the following criteria were excluded from the study: allergy or contraindication to any study medication; abnormal laboratory findings (total bilirubin or creatinine > 1.5 times the upper normal limit, or aspartate aminotransferase, and alanine aminotransferase or blood urea nitrogen > 2 times the upper normal limit); PPI or antibiotics taking within the preceding 4 weeks; pregnancy or lactating; female of childbearing age not using a medically accepted contraceptive method (menopausal women with more than twelve months of amenorrhea were regarded as nonchild bearing); presence of uncontrolled diabetes mellitus, hypertension, or hepatic disease; alcohol abuse; history of GI malignancy, except gastric adenoma or early gastric cancer resected by an endoscopic procedure within the preceding 5 years; previous stomach or esophageal surgery, such as gastrectomy or partial esophagostomy; and enrollment in another clinical research study within the preceding 30 days.

This clinical trial was approved by the DUIH Institutional Review Board (2014.121) and is registered at Clinical Trials.gov (NCT02401477). All subjects were provided with the study medication after written informed consent had been obtained.

### 2.2. Study Design

#### 2.2.1. Eradication Regimen

This was an open-labeled study and both the physicians and the subjects were not blinded to the study medication. Subjects received eradication therapy consisting of 2 drugs. The newly introduced ilaprazole (IL-Yang Pharmaceutical Co., Ltd., Seoul, Korea) as 40 mg tablets were taken twice a day, 1 hour before breakfast and dinner, approximately 12-hour interval, and 750 mg amoxicillin tablets were taken 4 times a day, 30 min after meals and before sleep. A 5- to 6-hour interval was recommended as the interval of administration. The total daily doses were 80 mg ilaprazole and 3,000 mg amoxicillin for 14 days. These drugs were offered in customized blister packs to assure that subjects kept to the study regimen.

**Figure 1:**
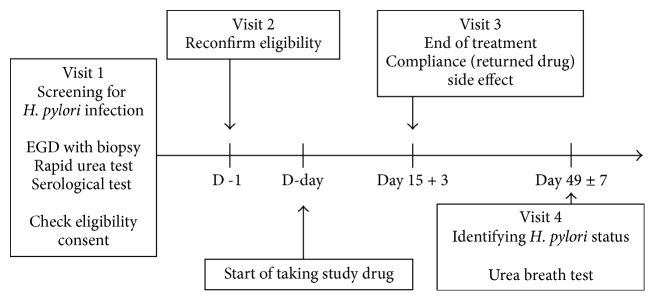
Flow chart of study methodology. EGD: esophagogastroduodenoscopy.

Subjects were requested to revisit the clinic after 14 days from the start of taking the study medication to determine compliance and any side effects. Subjects were instructed to return empty blister packs and all unused medications. Compliance was estimated using the remaining pills; taking less than 80% of all prescribed drugs was classified as poor compliance. Side effects were recorded as experienced symptoms after ingesting the drugs. PPIs and amoxicillin can cause unpleasant symptoms including nausea, vomiting, diarrhea, and abdominal pain. Side effects were assessed using a standard questionnaire, and any symptom that caused subjects to discontinue taking the medication was considered severe. Blood samples were taken to identify any systemic side effects, including drug-related liver function test abnormalities (Figure 1).

#### 2.2.2. Eradication Assessment

All subjects returned 4 to 6 weeks after treatment to assess eradication of the infection through a urea breath test (UBT). PPI use was prohibited during the 2-week period prior to assessment of therapeutic efficacy. Breath samples were obtained before and 15 min after oral administration of 100 mg urea labeled with ^13^C (UBIT; Korea Otsuka Pharmaceutical Co., Seoul, Korea), after an overnight fast. All samples were analyzed by infrared spectrophotometry (UBiT-IR300; Otsuka Electronics Co., Ltd., Osaka, Japan). The difference between the baseline 15-minute values was expressed as delta (*δ*) over baseline (DOB, *δ*‰), and DOB > 2.5‰ was considered positive [19]. We determined the eradication rate using an ITT analysis to estimate the practical efficacy and a PP analysis to estimate the biological efficacy. A success rate > 80% in the ITT analysis and > 90% in the PP analysis was defined as a suitable first-line eradication therapy [10, 20]. Entry of subjects into the study was designed to be ceased if it became clear that these rates could not be achieved to limit exposure of subjects to unnecessary risks according to the study termination criteria of 6 or more failures within 50 subjects or a success rate of less than 80% [21].

### 2.3. Statistical Analyses

Analyses were performed using SPSS for Windows ver. 20.0 software package (SPSS Inc., Chicago, IL, USA). Categorical variables were presented as number (percentage) and continuous variables as mean ± standard deviation. The eradication rate was calculated as a percentage of all enrolled subjects with a 95% confidence interval (CI). Categorical variables were compared among groups through the chi-square or Fisher's exact test as appropriate, and continuous variables were compared using Student's *t*-test. A *P* value of less than 0.05 was considered significant. All statistical methods were reviewed by the biostatistician (Chi Yeon Lim).

## 3. Results

### 3.1. Subject Characteristics

A total of 29 subjects were enrolled (17 male and 12 female; mean age, 47.9 ± 2 years). The* H. pylori* infection state was proven using rapid urease testing in 21 patients (72.4%) and using histology in 8 subjects (27.6%). Underlying gastric diseases diagnosed by endoscopy included gastritis (*n* = 27, 93.1%), gastric ulcer (*n* = 1, 4.3%), and duodenal ulcer (*n* = 1, 4.3%). Eight patients (27.6%) were current smokers, and 20 patients (69.0%) were social or heavy drinkers. All factors in the eradication success and failure groups are presented in Table 1. No differences were found in these factors between groups.

### 3.2. Eradication Rates

The success rate of* H. pylori* eradication was 23 of 29 or 79.3% (95% CI: 61.6–90.2) in the ITT analysis and 23 of 28 or 82.1% (95% CI: 64.4–93.2) in the PP analysis.

### 3.3. Compliance and Drug-Induced Side Effects

Compliance was found to be 96.6% (28/29). Only 1 patient did not take over 80% of the study drugs due to reasons unrelated to the medication or study itself. Side effects were reported by 3 subjects (10.3%): 2 cases of diarrhea and 1 of epigastric discomfort. These symptoms were mostly mild, and no subjects ceased treatment due to side effects. According to the blood test results, one patient exhibited a mild drug-related elevation of transaminases; however, these levels normalized 2 weeks later (Table 2).

## 4. Discussion

A large number of previous studies have found abundant evidence that* H. pylori* is etiologically related to GI diseases, including peptic ulcers, MALT lymphoma, and gastric cancer. The present recommendation is that an* H. pylori* infection should be eradicated with antibiotics in the above-mentioned diseases [22, 23].

Standard triple therapy based on PPI is the first-line treatment regimen, most commonly used worldwide. The eradication success rate using this therapy however has declined over the past decade and fails to reach even 80% in most studies in the central and southern European countries of Spain, France, Italy, and Turkey [24, 25]. These results are thought to be largely due to clarithromycin-resistant strains of* H. pylori* [26, 27], and, as such, the development of several alternative first-line therapeutic options is required. The most recent Maastricht IV/Florence Consensus Report recommended an alternative first-line therapy consisting of a bismuth-containing and/or nonbismuth quadruple therapy or sequential treatment to replace standard triple therapy in areas with a high resistance rate to clarithromycin (resistance rate over 15–20%) [28]. In addition to the above-mentioned method, high dose dual PPI plus amoxicillin or alternative antibiotics, such as rifabutin, furazolidone, and levofloxacin, has also been suggested as an alternative first-line therapy [29–32].

The present study evaluated effectiveness of high dose ilaprazole plus amoxicillin for* H. pylori* eradication. Ilaprazole is a new benzimidazole compound, so called third generation PPI, synthesized in Korea. In terms of pharmacokinetics, ilaprazole showed more prolonged plasma half-life time (8.1–10.1 hr) than prior PPIs (0.5–2 hr) [33] and it has been well known that ilaprazole is minimally metabolized by CYP2C19, main metabolic enzyme for first- and second-generation PPIs, so that lower variability of the medicinal effect was found among these PPI takers and there were no differences in mean intragastric pH for 24 hr, the percentage of time of a pH > 4, and healing rate of duodenal ulcer among the different phenotypes of CYP2C19 [34, 35]. In the present study, the eradication rate of* H. pylori* also had no difference among the different CYP2C19 phenotypes. To date, ilaprazole has several clinical data in spite of relatively short time of postmarket drug usage as compared with other PPIs. In the recent two studies, only studies that compared the gastric acid suppression of ilaprazole with that of other PPIs in healthy volunteers in China and Korea, ilaprazole exhibited significantly greater and prolonged suppression of gastric acid than omeprazole (ilaprazole 10, 20 mg versus omeprazole 20 mg) and esomeprazole (ilaprazole 20 mg, 40 mg versus esomeprazole 40 mg) [34, 36]. In the study for the efficacy of ilaprazole in duodenal ulcer, four randomized control trials displayed that ilaprazole was as effective as omeprazole [37].

The high dose dual therapy regimen in this study is based on two observations. Firstly, primary resistance to amoxicillin is usually low worldwide, and secondary amoxicillin resistance is also rare. The amoxicillin resistance rate of* H. pylori* is 0.5–2.2% but is as high as 17.8% in Africa [12, 28]. The bactericidal effect of amoxicillin against* H. pylori* is thought to be time-dependent, thus making more frequent doses better than less frequent doses [38, 39]. Secondly,* H. pylori* is thought to move into a nonreplicative but viable state when the intragastric pH is less than 6 but higher than 3. The bacteria move into the replicative state when the pH increases over 6 and in turn become susceptible to amoxicillin [40–42].

A study in Japan, using 10 mg rabeprazole and 500 mg amoxicillin, 4 times a day for 2 weeks, reported a cure rate of 90.9% (95% CI: 81.2–96.6) in the ITT analysis and 93.8% (95% CI: 84.8–98.3) in the PP analysis [43]. In addition, a study in China reported that eradication was achieved in 89.8% (95% CI: 82.1–87.6) of 117 subjects in the ITT analysis and 93.0% (95% CI: 86.4–99.6) in the PP analysis, using 20 mg rabeprazole twice and 1 g amoxicillin 4 times a day, for 2 weeks [44]. In this study, the eradication rate was 79.3% (23/29) in the ITT analysis and 82.1% (23/28) in the PP analysis. We administered 40 mg ilaprazole, which is 4 times the recommended dose for a duodenal ulcer [45]; yet the results did not meet our expectations. In a USA based study, using 40 mg esomeprazole plus 750 mg amoxicillin 3 times a day for 2 weeks, reported an ITT eradication rate of 72.2% (95% CI: 56–84) and 74.2% in the PP analysis (95% CI: 56–87) [46]. Another study in Korea using 30 mg lansoprazole plus 750 mg amoxicillin 3 times a day for 2 weeks showed that cure rates of based on ITT and PP analyses were 67.3% (95% CI: 58.3–76.3) and 74% (95% CI: 65.5–82.4), respectively [47]. The treatment success rate in the present study did not satisfy the requirements for first-line therapy according to the “report card” format with scales of A ≥ 95%, B = 90–94%, C = 85–89%, D = 81–84%, and F ≤ 80% from the ITT analyses and A = 95–100%, B = 90–94%, C = 86–89%, and F ≤ 85% from the PP analyses. The effectiveness grades based on the ITT and PP analyses were “F” and “unacceptable” [48]. Unfortunately, we found no higher eradication rate, even compared with the overall rate from a meta-analysis of standard triple therapy in Korea. This study set a goal of enrolling 54 subjects based on the sample size calculation prior to the study. We stopped enrolling additional subjects after the interim analysis for ethical reasons and to limit exposing the subjects to unnecessary risks according to the stop rule (6 or more failures within 50 subjects or an eradication rate of less than 80%).

Compliance of the proposed eradication regimen in the present study was high and side effects were mild and minimal. No subjects stopped taking the prescribed drugs due to side effects, and no significant difference was found in the prevalence of side effects between the eradication success and failure groups. Therefore, these factors apparently did not influence the low eradication rate in this study. Smoking has previously been considered a significant factor related to treatment failure of dual therapy [49]. Smoking lowers intragastric pH by raising acid secretion and decreasing pancreatic and duodenal bicarbonate secretion [50–52]. Although our study population was small, we found no association between smoking and eradication rate. We also hypothesized that drinking could affect drug compliance, but drinking status did not affect the eradication rate. Consequently, compliance, side effects, smoking, and drinking states were not associated with the eradication rate in this study.

This study had several limitations. We did not measure 24 hr intragastric pH during the study, so we were unable to ascertain whether intragastric pH was maintained over 6 and for how long. However, we did calculate the dose of ilaprazole to be equivalent to the lansoprazole dose that has been shown to maintain the intragastric pH over 6.

In conclusion, high dose ilaprazole twice daily and amoxicillin 4 times daily was ineffective as a first-line therapy to eradicate* H. pylori* in Korea. The best reported results have been obtained by administering a PPI and amoxicillin 4 times a day [13]. It seems prudent in the future to start with 4 times a day PPI and amoxicillin administration or alter the timing of PPI administration based upon the intragastric pH measurements and to test for amoxicillin resistance.

## Figures and Tables

**Table 1:** Baseline characteristics of the study population.

Characteristics	Eradication success^*∗*^ *n* = 23	Eradication failure *n* = 6	*P* value
Gender, *n* (%)			0.20
Male	15 (65.2)	2 (33.3)	
Female	8 (34.8)	4 (66.7)	
Age, years, mean ± SD	47.5 ± 10.6	49.3 ± 7.1	0.63
Underlying gastric disease, *n* (%)			
Gastritis	21 (91.3)	6 (100.0)	1.00
Gastric ulcer	1 (4.3)	0 (0.0)	1.00
Duodenal ulcer	1 (4.3)	0 (0.0)	1.00
Smoking, *n* (%)			
Current smoker	7 (30.4)	1 (16.7)	0.65
Ex-smoker	5 (21.7)	0 (0)	0.55
Never smoker	11 (47.9)	5 (83.3)	0.18
Alcohol, *n* (%)			
Nonalcohol drinker	6 (26.1)	3 (50.0)	0.55
Social drinker^†^	14 (60.9)	2 (33.3)	0.25
Heavy drinker^‡^	3 (13.0)	1 (16.7)	0.36
Compliance, *n* (%)			0.20
Good	23 (100)	5 (83.3)	
Bad^§^	0 (0)	1 (16.7)	
Side effect, *n* (%)	3 (13.0)	0 (0)	1.00

^*∗*^ *Helicobacter pylori* eradication rate was 79.3% based on the intention-to-treat analysis. ^†^Drinking fewer than 3 bottles/week, ^‡^drinking more than 3 bottles/week, and ^§^taking less than 80% of all study drugs. The *P* value was derived from Student's *t*-test for age variable, and the *P* values for the other variables were derived from the chi-square or Fisher's exact test. SD: standard deviation.

**Table 2:** Side effects of high dose ilaprazole plus amoxicillin therapy.

Side effects	Number of subjects (%) *n* = 29
Nausea	0 (0.0%)
Diarrhea	2 (6.9%)
Vomiting	0 (0.0%)
Abdominal pain	0 (0.0%)
Metallic taste	0 (0.0%)
Epigastric soreness	1 (3.4%)
Drug related LFT abnormality	1 (3.4%)

LFT: liver function test.
